# Triphenylborane in Metal-Free Catalysis

**DOI:** 10.3390/molecules28031340

**Published:** 2023-01-31

**Authors:** Suresh Mummadi, Clemens Krempner

**Affiliations:** 1 Department of Natural Sciences, The University of Virginia’s College, Wise, VA 24293-4400, USA; 2 Department of Chemistry and Biochemistry, Texas Tech University, Lubbock, TX 79409-1061, USA

**Keywords:** triphenyl borane, metal-free catalysis, Lewis acid, frustrated Lewis pair, cycloaddition, hydrogenation, hydrosilylation, polymerization, carbon dioxide

## Abstract

The development and application of new organoboron reagents as Lewis acids in synthesis and metal-free catalysis have dramatically expanded over the past 20 years. In this context, we will show the recent uses of the simple and relatively weak Lewis acid BPh_3_—discovered 100 years ago—as a metal-free catalyst for various organic transformations. The first part will highlight catalytic applications in polymer synthesis such as the copolymerization of epoxides with CO_2_, isocyanate, and organic anhydrides to various polycarbonate copolymers and controlled diblock copolymers as well as alternating polyurethanes. This is followed by a discussion of BPh_3_ as a Lewis acid component in the frustrated Lewis pair (FLP) mediated cleavage of hydrogen and hydrogenation catalysis. In addition, BPh_3_-catalyzed reductive N-methylations and C-methylations with CO_2_ and silane to value-added organic products will be covered as well along with BPh_3_-catalyzed cycloadditions and insertion reactions. Collectively, this mini-review showcases the underexplored potential of commercially available BPh_3_ in metal-free catalysis.

## 1. Introduction

Last year marked the 100th anniversary of the discovery of triphenyl borane, BPh_3_, the first isolated triaryl borane by E. Krause and R. Nitsche [1]. The authors obtained BPh_3_ as a crystalline solid through the treatment of BF_3_ with excess phenyl magnesium bromide followed by distillation under vacuum. About 50 years later, its solid-state structure was determined by single crystal X-ray crystallography [2]. The results revealed a trigonal planar coordination environment for boron with the three phenyl rings being tilted by about 30° toward the plane.

BPh_3_ has found widespread application as a promoter in the hydrocyanation of olefins in the presence of Ni complexes and is used industrially by Du Pont for its hydrocyanation of butadiene to adiponitrile, a nylon intermediate [3]. In addition, BPh_3_ has been employed extensively as a diphenyl boryl transfer agent in the synthesis of boron-containing heterocyclic materials with remarkable photophysical properties, such as electro- and photoluminescence and aggregation-induced emission (AIE) [4,5,6,7,8,9,10,11,12,13,14,15,16,17]. BPh_3_ is a relatively weak Lewis acid that forms Lewis acid–base adducts with pyridine and a wide variety of aliphatic amines [18]. These stable adducts have found use as catalysts for the polymerization of acrylic esters [3], as antifouling reagents in marine environments [19,20,21] and as agrochemical fungicides [22,23,24].

Despite its early discovery and structural simplicity, BPh_3_ has not risen to the same prominence amongst scientists in academia as has its ingenious perfluorinated and highly Lewis acidic counterpart, B(C_6_F_5_)_3_, first reported in 1963 [25]. Perhaps one major factor that has caused BPh_3_ to lag behind B(C_6_F_5_)_3_, particularly in the emerging field of metal-free Lewis acid catalysis, is its comparably low Lewis acidity [26]. As high Lewis acidity is often key to the activation and rapid catalytic transformation of organic substrates, it is not a surprise that B(C_6_F_5_)_3_ has been at the forefront of current research in metal-free catalysis [27,28,29,30,31,32,33,34,35,36]. However, as the development and application of new boron Lewis acids have expanded at an amazing pace over the past 20 years, it has also become apparent that tuning the Lewis acidity of the catalyst can be an important factor in achieving new modes of substrate activation and selectivity. A prime example of this development has been the application of frustrated Lewis pairs (FLPs) [37,38,39] in catalytic hydrogenations of unsaturated organic molecules. Work from various groups has shown that weaker Lewis acid components may exhibit better performances, improved functional group tolerances, or different selectivities [40,41,42,43,44,45].

In this context, this mini-review aims to showcase the underexplored potential of commercially available and weakly Lewis-acidic BPh_3_ in metal-free catalysis. Particular emphasis is given to the role of BPh_3_ as a catalyst in polymer synthesis, in frustrated Lewis pair (FLP) mediated hydrogen cleavage, and hydrogenation catalysis as well as transformations of CO_2_ to value-added organic products and Lewis acid catalyzed cycloadditions and insertion reactions.

## 2. BPh_3_ in Polymerization Catalysis

**Scheme 1:**
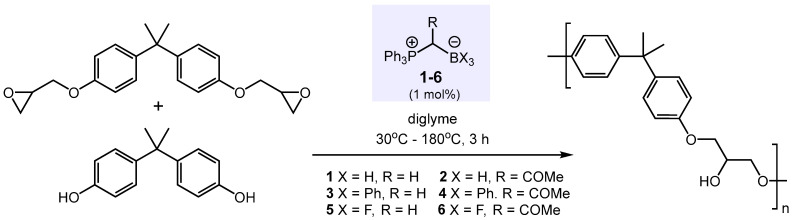
Catalytic copolymerization of bisphenol A and bisphenol A diglycidyl ether.

In developing effective “latent catalysts” for the curing of epoxy resins that overcome issues associated with previously utilized systems, such as poor solubility and hygroscopicity, Endo and co-workers studied Lewis pairs of general formula Ph_3_PCHRBX_3_, where X = H, Ph, F (Scheme 1) [46,47]. Most of these air- and moisture-stable zwitterionic phosphonium borates **1**–**6**, derived from reactions of the respective boranes with phosphonium ylides, proved to be active pre-catalysts in the co-polymerization of bisphenol A and bisphenol A diglycidyl ether at high temperatures (Scheme 1). The pre-catalyst activity at 120 °C was found to be in the following order: **4** > **2** > **1** > **3** > **5** > **6**. The fact that BPh_3_-adduct **4** and BH_3_-adduct **2** converted the bisphenol A diglycidyl ether within 3 h to ca. 90% and 70%, respectively, whereas BF_3_-adduct **6** was essentially inactive, impressively shows the importance of steric and electronic parameters in the design of Lewis pairs as active polymerization catalysts.

**Scheme 2:**
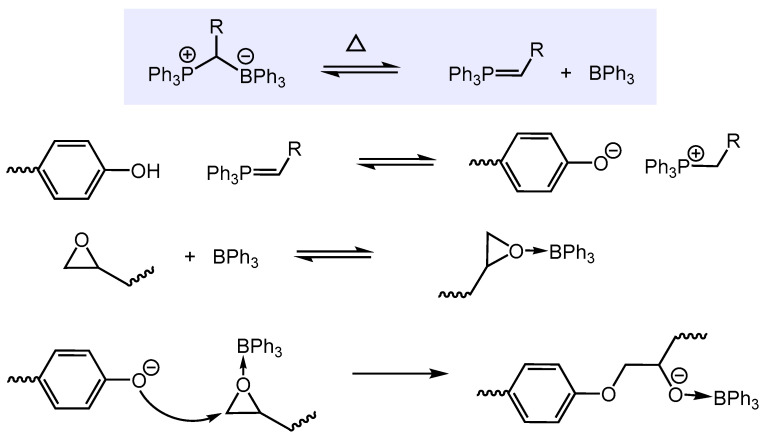
Proposed mechanistic steps of the catalytic copolymerization of bisphenol A and bisphenol A diglycidyl ether.

The key to the catalytic activity of the phosphonium borate catalysts **1**-**6** was assumed to be their thermally induced dissociation to BX_3_ and Ph_3_P=CHR, with the degree of dissociation being a sensitive function of temperature as well as the strength of the C-B bond (Scheme 2). The significantly better performances of the BPh_3_-ylide and BH_3_-ylide adducts **4** and **2** compared to the BF_3_-ylides **5** and **6** have been attributed to their weaker B-C bonds due to the lower Lewis acidity of BPh_3_ and BH_3_. Upon dissociation of the adduct, the resulting phosphonium ylide serves as an initiator deprotonating the phenolic OH group of bisphenol A to generate a phenoxide anion. BX_3_ activates, as a Lewis acid, the epoxide functionality, facilitating its ring-opening via nucleophilic attack of the phenoxide anion. Whether BX_3_ remains to be intact as a catalyst during the course of the reaction is questionable given the harsh conditions and the Bronsted acidity of bisphenol A.

The past decade has witnessed increasing interest in the metal-free catalyzed formation of cyclic organic carbonates and polycarbonates from epoxide monomers and CO_2_ as an alternative to metal-based catalysts [48,49,50,51]. Recent reports from Feng [50] and Darensbourg [51] demonstrated that metal-free Lewis pairs can be utilized as effective catalysts in the copolymerizing of propylene oxide (PO) with CO_2_ or carbonyl sulfide (COS). The catalyst systems comprised triethyl borane as a Lewis acid, and amines, onium salts, or alkoxides as Lewis bases.

**Scheme 3:**
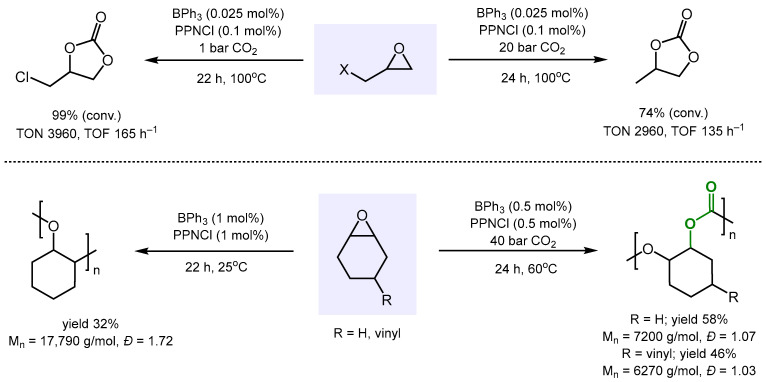
BPh_3_-catalyzed formation of cyclic carbonates and polycarbonates.

The Kerton group investigated the activity of BPh_3_ and B(C_6_F_5_)_3_ as catalysts and bis(triphenylphosphine)iminium chloride (PPNCl) as a co-catalyst for the reaction of propylene oxide (PO) and CO_2_ at 100 °C (Scheme 3) [52]. Both were active catalysts in generating propylene carbonate under identical conditions. These findings were in stark contrast with previous work using triethyl borane as the catalyst, which produced poly(propylene carbonate) [50]. It is worthwhile noting that based on initial rate measurements, the catalyst system BPh_3_/PPNCl was more than five times faster than B(C_6_F_5_)_3_/PPNCl. This is most likely due to the significantly higher Lewis acidity of B(C_6_F_5_)_3_, which binds much more strongly to the Cl^-^ anion of co-catalyst PPNCl than BPh_3_ does. Consistent with kinetic studies revealing that lowering the CO_2_ pressure increases the reaction rate, BPh_3_ catalyzed the reaction of epichlorohydrin with CO_2_, even under atmospheric CO_2_ pressure, to give the respective cyclic carbonate as the sole product.

In addition, BPh_3_ was found to catalyze the copolymerization of CO_2_ with cyclohexene oxide (CHO) and vinyl cyclohexene oxide (VCHO), respectively, at low catalyst loadings. Polycarbonates with high numbers of averaged molecular weights and excellent polydispersities were obtained. In the absence of CO_2_, BPh_3_ catalyzed the ring opening of CHO to give the epoxide homopolymer (Scheme 3).

**Scheme 4:**
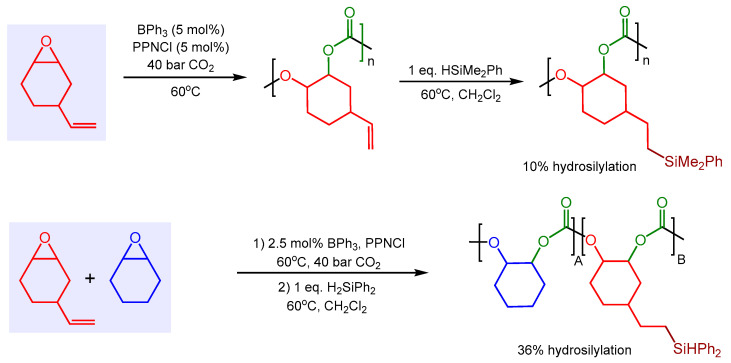
Sequential BPh_3_-catalyzed copolymerization and hydrosilylation.

In a follow-up study, Kerton and co-workers investigated the ability of BPh_3_ to catalyze the addition of Si–H groups onto a vinyl-substituted polycarbonate [53]. In a ‘one-pot’ sequence, BPh_3_ catalyzed both reactions: the copolymerization of VCHO and CO_2_ to polycarbonate followed by the hydrosilylation of the vinyl groups in the polymer with phenyl dimethyl silane to give side-chain silylated polycarbonate (Scheme 4). Perhaps for steric reasons, the degree of hydrosilylation of polycarbonate was only 10%. However, when using a polycarbonate terpolymer derived from CHO, VCHO, and CO_2_, the degree of hydrosilylation could be increased to 36%, in this case with H_2_SiPh_2_ as the hydrosilylation reagent.

**Figure 1:**
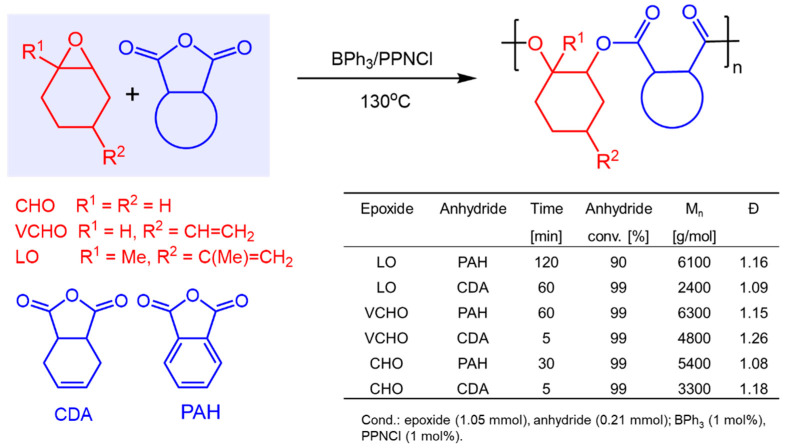
BPh_3_-catalyzed copolymerization of organic anhydrides and epoxides.

In 2021, Kerton et al. disclosed the BPh_3_/PPNCl catalyzed copolymerization of organic anhydrides and epoxides [54]. Cyclohexene oxide (CHO), vinyl cyclohexene oxide (VCHO) and also limonene oxide (LO) could be polymerized with phthalic anhydride (PAH) and *cis*-4-cyclohexene-1,2-dicarboxylic anhydride (CDA), respectively, to give perfectly alternating copolymers with excellent polydispersities (Figure 1).

**Scheme 5:**
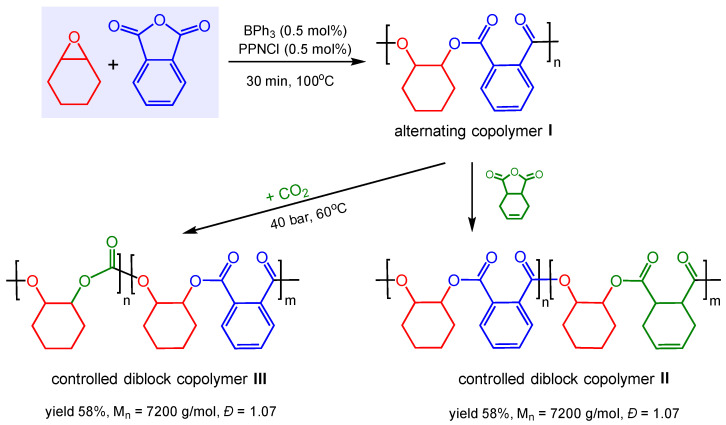
BPh_3_-catalyzed copolymerization to alternating copolymers and controlled diblock copolymers.

In addition, the authors discovered that these perfectly alternating co-polymers can be further polymerized to form controlled diblock copolymers using BPh_3_/PPNCl as the catalyst system. For example, PAH was first allowed to react with excess CHO to generate copolymer **I**. Once the full conversion of PAH was achieved, the second anhydride, CDA, was added and allowed to react to completion, resulting in the selective formation of controlled diblock copolymer II (Scheme 5). Similarly, by sequentially adding CO_2_ to the in situ generated copolymer I, controlled diblock copolymer III could be obtained again with high selectivity and excellent polydispersities. It is worthwhile noting that the markedly stronger Lewis acid B(C_6_F_5_)_3_ was not an active catalyst either for epoxide/anhydride copolymerization or for epoxide/anhydride/CO_2_ block copolymerizations. However, it was found that when B(C_6_F_5_)_3_ was added to diblock copolymer III, the carbonate block of the polymer was depolymerized to give the respective cyclic carbonate.

**Scheme 6:**
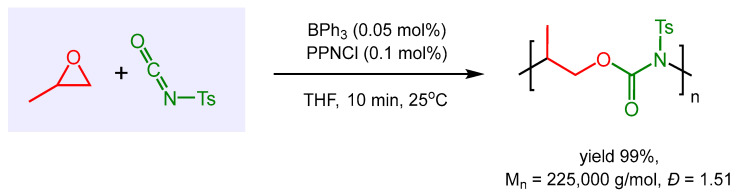
BPh_3_-catalyzed copolymerization of propylene oxide (PO) and p-tosyl isocyanate (TSI).

Gnanou, Feng, and co-workers investigated triethyl borane (BEt_3_) catalyzed copolymerizations of epoxides with organic isocyanates to polyurethanes [55], challenging transformations due to the propensity of most isocyanates to undergo side-reactions such as homopolymerization, cyclotrimerization and [2 + 3] cycloaddition with epoxides. Strongly electron-withdrawing p-tosyl isocyanate (TSI) was found to be the most suitable substrate selectively undergoing copolymerization with a range of epoxides to form almost perfectly alternating polyurethanes (less than 1% ether linkages in the polymer) in high yields and purities. Interestingly, when investigating the impact of the Lewis acidity of the borane catalyst on the rate of propylene oxide/TSI copolymerization, it was found that with BPh_3_, a stronger Lewis acid than BEt_3_, the reaction occurred explosively with the release of large amounts of heat. Astonishingly, with very low catalyst loadings of 0.05 mol% BPh_3_ and 0.1 mol% PPNCl, complete polymerization was accomplished within 10 min with turnover frequencies of over 10,000 h^−1^, and a remarkably high number of averaged molecular weight (M_n_ = 225,000 g/mol), exclusively alternating polyurethane structure and a polydispersity of 1.51. Unfortunately, a substrate scope with this highly active and selective catalyst system was not investigated (Scheme 6).

## 3. BPh_3_ as an FLP Component in Hydrogenation Catalysis

Stephan and co-workers’ discovery that Lewis acidic boranes can actively participate in the heterolytic cleavage of H_2_ via a frustrated Lewis pair (FLP) approach [37,38,39] opened the door to the design of metal-free catalysts for the hydrogenation of unsaturated organic substrates [56,57,58,59,60]. Briefly, FLPs comprise sterically encumbered Lewis acid–base pairs, unable to form Lewis acid–base adducts due to unfavorable steric interactions. As a result, Lewis acidity and basicity of the individual FLP components remain unquenched, thus enabling the heterolytic cleavage of H_2_, a key step in catalytic hydrogenation reactions [41,42,43,44,45]. Typically, active FLP catalysts are comprised of strongly Lewis acidic, often highly fluorinated or chlorinated aryl boranes coupled with relatively weak N- or P-containing Lewis bases. Theoretical studies concerning the thermodynamic feasibility of the FLP-mediated H_2_ cleavage, however, suggested that weak Lewis acids could be active as well, provided that a sufficiently strong base is present [61]. In this context, it is worthwhile noting that Stephan, in one of his seminal papers [38], disclosed the stochiometric H_2_ cleavage utilizing the FLPs B(C_6_F_5_)_3_/P(Bu^t^)_3_ and BPh_3_/P(Bu^t^)_3_. The former FLP with B(C_6_F_5_)_3_ (ΔH_HA_ = −112 kcal/mol) quickly and quantitatively generated the phosphonium borate salt [HB(C_6_F_5_)_3_][HP(Bu^t^)_3_] at room temperature, whereas with less acidic BPh_3_ (ΔH_HA_ = −74.4 kcal/mol) salt [HBPh_3_][HP(Bu^t^)_3_] was formed in only 33% yields after 24 h. However, theoretical calculations by Papai and co-workers appear to contradict the effectiveness of BPh_3_/P(Bu^t^)_3_ to cleave H_2_ as the reaction was calculated to be endergonic (ΔG_R_ = +18.2 kcal/mol), while for B(C_6_F_5_)_3_/P(Bu^t^)_3_ the reaction with H_2_ was exergonic (ΔG_R_ = −14.7 kcal/mol) [61].

**Scheme 7:**
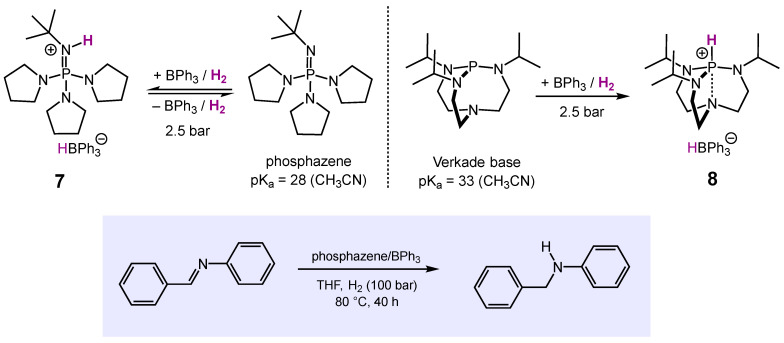
FLP-mediated stochiometric H_2_-cleavage and catalytic hydrogenation of N-benzylidene aniline.

Building on these and other results [62,63], Krempner et al. employed the bulky organosuperbases Verkade base (p*K*_a_ = ~33 in CH_3_CN) and phosphazene (p*K*_a_ ~28 in CH_3_CN) in combination with BPh_3_ (Scheme 7) [64]. Upon exposure to H_2_, both FLPs instantly generated the corresponding borate salts **7** and **8** in yields of 71% and 85%, resp. Solutions of **8** appeared to be thermally stable, while **7** in solution quickly released H_2_ when heated to 60 °C, indicating reversibility of heterolytic hydrogen cleavage, which is key to the development of effective hydrogenation catalysts. In fact, BPh_3_/phosphazene was demonstrated to be an active FLP catalyst system in the quantitative hydrogenation of the N-benzylidene aniline to N-benzyl aniline in THF as solvent.

**Scheme 8:**
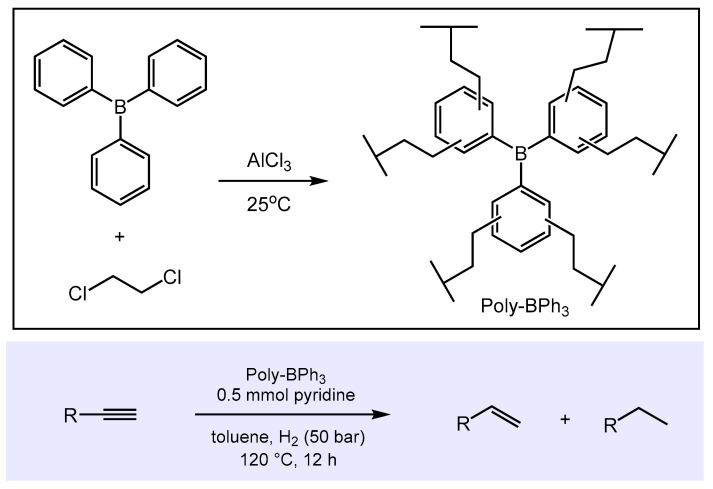
Synthesis and catalytic activity of Poly-BPh_3_ in the FLP-mediated semi-hydrogenation of alkynes to alkenes.

An interesting extension of this concept was recently introduced by Hu and co-workers for the FLP-catalyzed hydrogenation of alkynes [65]. Because the molecular FLP BPh_3_/pyridine thermally degraded during hydrogenation of phenylacetylene (conversion 12% after 12 h at 120 °C and 50 bar H_2_), the group developed a polymeric Lewis acid based on BPh_3_. The synthesis is illustrated in Scheme 8 and involves a classical Friedel–Crafts reaction of BPh_3_ with 1,2-dichloroethane in the presence of AlCl_3_.

This polymeric Lewis acid, Poly-BPh_3_, combined with pyridine as the Lewis base was shown to be active in the semi-hydrogenation of a variety of aliphatic and aromatic terminal alkynes at 120 °C to preferentially give the corresponding alkenes. The Lewis acid component of this FLP, Poly-BPh_3_, is reusable without loss of catalytic activity after being recovered from the reaction mixture, and appears to be thermally and hydrolytically remarkably stable.

**Scheme 9:**
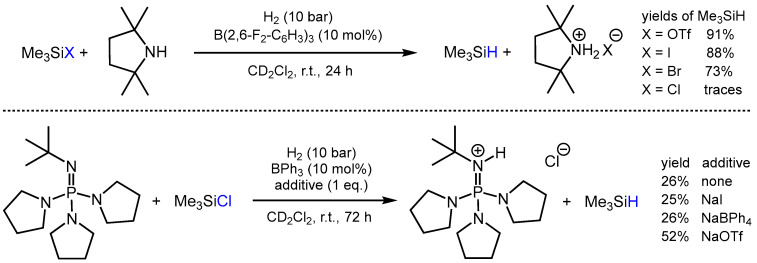
FLP-catalyzed hydrogenation of Me_3_SiX to Me_3_SiH.

Recent developments concerning iridium- and ruthenium-catalyzed hydrogenations of organosilicon compounds [66,67,68,69,70] motivated the Cantat group to design FLP catalysts for the metal-free hydrogenation of various organosilanes with Si-X bonds (X = OTf, I, Br, Cl) [71]. After extensive screening, B(2,6-F_2_-C_6_H_3_)_3_ as the Lewis acid combined with stochiometric amounts of 2,2,6,6-tetramethylpiperidine (TMP) as the base was identified as the most effective FLP catalyst for the hydrogenation of Me_3_SiX (X = OTf, I, Br) to Me_3_SiH (Scheme 9). Employing the same FLP catalyst, Et_3_SiOTf, Ph_3_SiOTf, and (Pr^i^)_2_Si(OTf)_2_ could be hydrogenated to the corresponding hydrosilanes in good yields as well. However, irrespective of the base used, B(2,6-F_2_-C_6_H_3_)_3_ was not able to convert the more challenging substrate Me_3_SiCl to Me_3_SiH, primarily because of the comparably stronger Si-Cl bond and lower hydricity of the respective borohydride anion, HB(2,6-F_2_-C_6_H_3_)_3_^–^, formed upon H_2_-splitting. Building on the idea of favoring the thermodynamics by increasing the hydricity of the borohydride, less Lewis acidic BPh_3_ was tested in combination with stronger base phosphazene to ensure H_2_-cleavage. With this FLP in hand, 28% of Me_3_SiCl were converted after 72 h to Me_3_SiH in yields of 26%. Moreover, upon adding the chloride abstracting additive NaOTf, the yields of Me_3_SiH were further increased to a respectable 52% (Scheme 9).

**Scheme 10:**
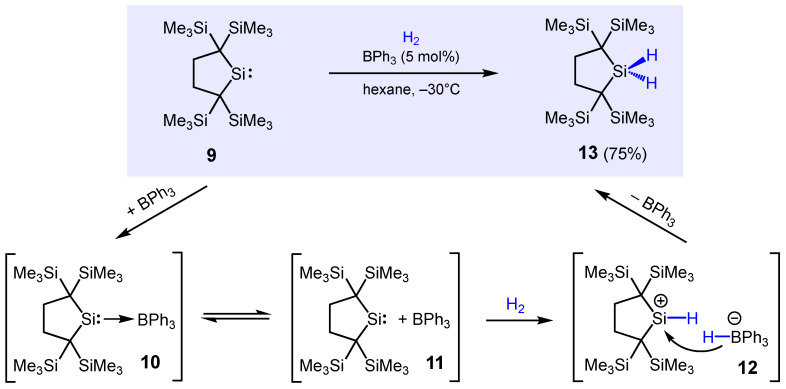
BPh_3_-catalyzed hydrogenation of a silylene and proposed mechanism of formation.

Silylenes, divalent silicon species of general formula R^1^R^2^Si, are known to exhibit amphoteric behavior, capable of serving either as Lewis acids or Lewis bases. In this respect, Kira, Mueller, and co-workers investigated the potential of sterically overcrowded silylene **9** to be an active component in the heterolytic cleavage of H_2_ via an FLP approach (Scheme 10) [72]. It was found that **9** not only actively engaged in H_2_ splitting, it was also fully hydrogenated to hydrosilane **13** in isolated yields ranging from 75–84% with catalytic amounts of either Lewis base (PPh_3_, NPh_3_, PEt_3,_ and NEt_3_) or Lewis acids such as BEt_3_ and BPh_3_. NMR spectroscopic investigations in solution showed that neither PPh_3_ nor BPh_3_ forms a classical Lewis acid–base adduct, indicative of FLP formation in both cases. Nonetheless, when H_2_ was introduced into the solution, rapid formation of hydrosilane **13** occurred. Based on DFT calculations, a mechanism was proposed in which BPh_3_ initially forms the weak complex **10** with the silylene, which is stabilized by ca. −7 kcal/mol compared to the starting materials. Subsequent heterolytic cleavage of H_2_ leads to the formation of silylium borate **12**; its formation is slightly endothermic (+14 kcal/mol). Finally, the hydride is transferred from the borohydride to the silylium cation to give the final product **2**, a strongly exothermic process (−37 kcal/mol). It should be noted that strong Lewis acid B(C_6_F_5_)_3_ was not capable of hydrogenating silylene **9**, instead, degradation occurred.

## 4. Hydrosilylation Catalysis

**Scheme 11:**
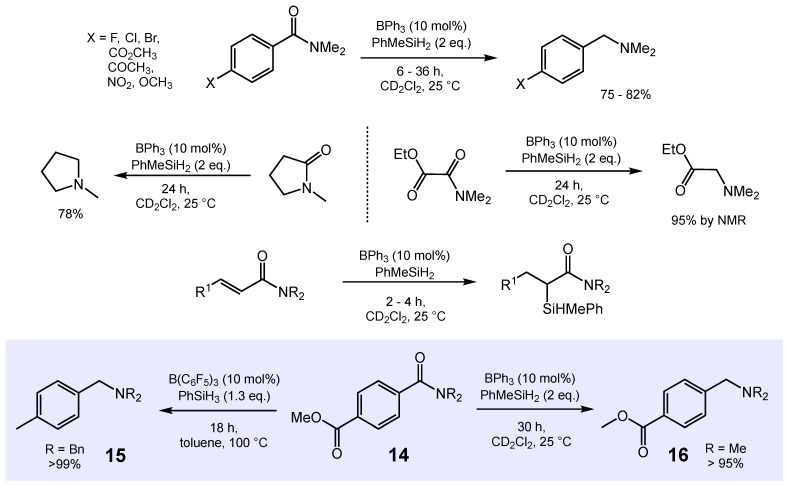
Catalytic hydrosilylation of organic amides to amines.

Arguably, one of the most challenging metal-free catalyzed reductive transformations represents the reduction of organic amides to amines [73,74,75]. In 2016, Okuda and co-workers reported the highly selective hydrosilylation of a variety of aromatic and aliphatic tertiary amides to the corresponding amines with 10 mol% BPh_3_ as the catalyst system and 2 eq. of PhMeSiH_2_ as the reducing agent (Scheme 11) [76].

Aromatic and aliphatic tertiary amines were obtained in good to excellent isolated yields. In contrast, α,β-unsaturated amides underwent hydrosilylation of the olefinic groups (C=C) to give the respective α-silylated amides. In addition, this process showed a good functional group tolerance with additives such as acetophenone, ethyl acetate, N-benzylidene aniline, *tert*-butyl isocyanate, and thiophene not inhibiting the activity of the catalyst. Only in the case of benzaldehyde and ethanol, hydrosilylation and dehydrosilylation, respectively, occurred prior to amide reduction. The addition of pyridine deactivated the catalyst due to irreversible Lewis acid–base adduct formation with BPh_3_. The BPh_3_-catalyzed amide hydrosilylations described by Okuda’s group appear to be superior in terms of both functional group tolerance and chemo-selectivity when compared with other metal-free catalysts reported in the literature [74,75]. For example, B(C_6_F_5_)_3_ catalyzes the reduction of both functional groups of **14**, the ester as well as the amide, to give benzylamine **15**. In contrast, BPh_3_ reduces the amide group while keeping the ester group intact leading to the selective formation of the ester-functionalized benzylamine **16** (Scheme 11) [74].

**Scheme 12:**
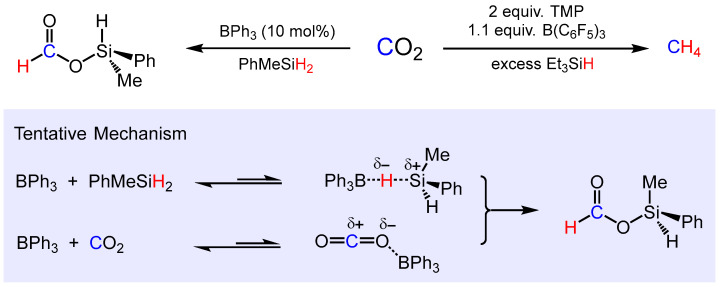
Lewis acid catalyzed hydrosilylation of CO_2_ and proposed mechanism of silyl formate formation (TMP = 2,2,6,6-tetramethylpiperidine).

The same group reported the BPh_3_-catalyzed hydrosilylation of CO_2_ (Scheme 12) [77]. This approach enabled the highly selective formation of silyl formates with various hydro silanes such as PhSiH_3_, PhMeSiH_2_ or Et_3_SiH in polar solvents such as acetonitrile, nitromethane and propylene carbonate. No turnover was observed in less polar and non-polar solvents such as benzene, toluene, tetrahydrofuran (THF) and CH_2_Cl_2_. Mechanistically the BPh_3_-catalyzed hydrosilylation of CO_2_ is suggested to proceed via the dual activation of CO_2_ and organosilane by BPh_3_, where polar solvents with high dielectric constants stabilize the partially charged transient species (Scheme 11). Note that strongly Lewis acidic B(C_6_F_5_)_3_ itself was not capable of catalyzing the hydrosilylation of CO_2_, while as the FLP component in combination with tetramethyl piperidine (TMP) as a Lewis base and with an excess of triethylsilane CO_2_ was quantitatively reduced to give CH_4_ [78].

**Scheme 13:**
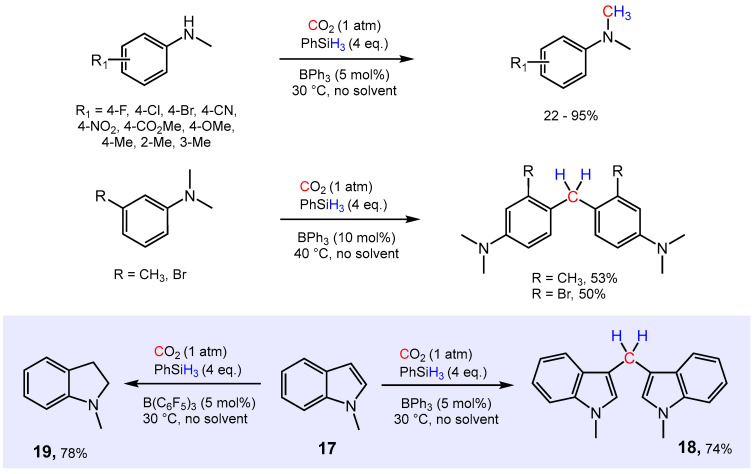
BPh_3_-catalyzed *N*-methylation and C-methylenation of secondary and tertiary aromatic amines.

More recently, Ema and co-workers reported the utilization of CO_2_ as C1-feedstock for the reductive methylation of *secondary* and tertiary aromatic amines derivatives, employing PhSiH_3_ as a reducing agent and BPh_3_ as the catalyst (Scheme 13) [79,80]. The N-methylation of *sec*-amines was achieved with 1 atm of CO_2_, 4.0 equivalents of PhSiH_3_ and 5 mol% of BPh_3_ under solvent-free conditions at 30 °C. The process tolerates various functional groups such as halides, nitrile, nitro, ester and alkoxy groups. On the other hand, C-methylenation was observed with tertiary aromatic amines to give the corresponding diarylmethanes in moderate yields at 40 °C. Note that B(C_6_F_5_)_3_ was inactive even at higher temperatures in any of these transformations, probably due to irreversible Lewis acid–base adduct formation. Similar to what was seen for the reaction of tertiary aromatic amines, 1-methylindole at 30°C converted to 3,3′-methylenebis(1-methylindole) **18** in good yields. On the contrary, B(C_6_F_5_)_3_ catalyzed the formal hydrogenation of the olefinic bond of N-methyl indole to selectively give N-methyl dihydroindole **19**.

**Scheme 14:**
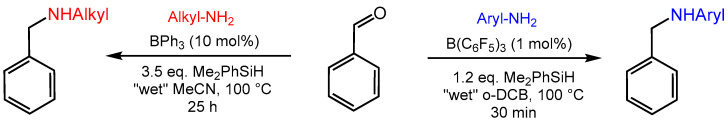
Triaryl borane catalyzed reductive amination of aldehydes.

Motivated by the moderate water tolerance of B(C_6_F_5_)_3_ in catalytic hydrogenation reactions [81], Ingleson and co-workers disclosed the triaryl borane catalyzed reductive aminations of aldehydes to aromatic and aliphatic amines using Me_2_PhSiH as the reducing agent [82,83]. Notably, the employed catalysts BPh_3_ and B(C_6_F_5_)_3_ showed strikingly different selectivities for aliphatic and aromatic amine substrates (Scheme 14). With B(C_6_F_5_)_3_ as the catalyst, the amine substrates were limited to aryl amines (pK_a_ of the conjugate acid < 12), while with weaker Lewis acidic BPh_3_, the more basic alkyl amines (pK_a_ of the conjugate acid > 16) performed well with excellent conversions and yields. These findings were attributed to the different interactions of the amine base with the intermediately formed water adducts H_2_O→BPh_3_, and H_2_O→B(C_6_F_5_)_3_. Thus, with more basic alkyl amines, irreversible deprotonation of the highly acidic adduct H_2_O→B(C_6_F_5_)_3_ (pK_a_ = 8.4 in CH_3_CN) occurred, resulting in the degradation of the catalyst. BPh_3_, on the other hand, undergoes rapid protodeboronation in the presence of the more acidic aryl amines. It is worth noting that the in situ generated Lewis acid B(3,5-Cl_2_-C_6_H_3_)_3_, whose Lewis acidity is in between B(C_6_F_5_)_3_ and BPh_3_, was capable of catalyzing the reductive amination with both aryl and alkyl amines. However, a detailed substrate scope was not explored with B(3,5-Cl_2_-C_6_H_3_)_3_ as the catalyst.

## 5. Miscellaneous

**Scheme 15:**
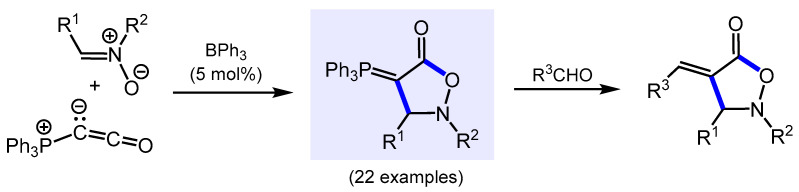
BPh_3_-catalyzed formal [2 + 3] cycloaddition of Ph_3_PCCO with nitrones to 5-isoxazolidinone with exocyclic phosphonium ylide moiety and their Wittig reaction.

Krempner and co-worker reported the Lewis acid catalyzed [2 + 3] cycloaddition of Bestmann’s ylide, Ph_3_P=C=C=O, with nitrones to produce a variety of previously unknown 5-isoxazolidinones with exocyclic phosphonium ylide functionality in excellent isolated yields [84]. Subsequent quenching with reactive aldehydes via a classical Wittig reaction gave access to 5-isoxazolidinones with exocyclic double bonds (Scheme 15).

**Scheme 16:**
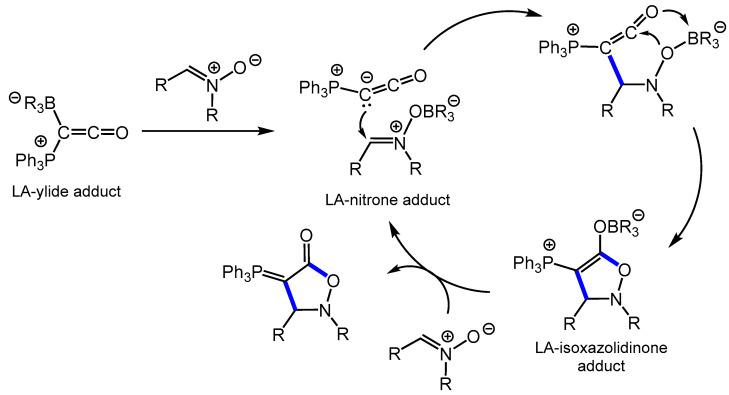
Proposed mechanism of the BPh_3_-catalyzed formal [2 + 3] cycloaddition of Ph_3_PCCO with nitrones (LA = Lewis acid).

Of the boron-containing Lewis acid catalysts tested, BPh_3_ proved to be most effective in providing the cycloaddition product in almost quantitative yields within 8 h at room temperature. Weaker Lewis acids such as BEt_3_, BMes_3_ and B(OMe)_3_ were inactive even at elevated temperatures, while B(C_6_F_5_)_3_, the strongest amongst the Lewis acids studied, required 80 °C and 16 h to quantitatively produce the cycloaddition product. The authors proposed a mechanism (Scheme 16) in which the borane catalyst activates the nitrone via Lewis acid–base interactions, which facilitates the nucleophilic attack of the ylidic carbon of Ph_3_PCCO resulting in the cyclized borane-5-isoxazolidinone adduct from which the 5-isoxazolidinone is liberated via borane–nitrone adduct formation. The higher activity of the BPh_3_ over its more acidic counterpart B(C_6_F_5_)_3_ is attributed to the latter forming stable adducts with both Ph_3_PCCO and the nitrone, while BPh_3_ does not [85].

**Scheme 17:**
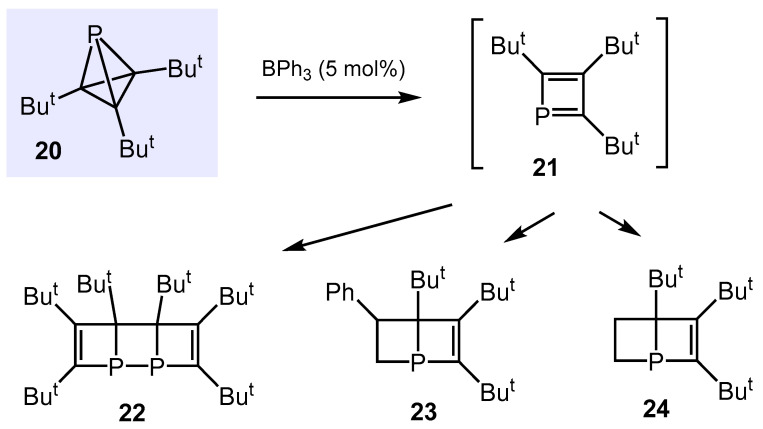
BPh_3_-catalyzed reactions of tri-*tert*-butylphosphatetrahedrane.

Recently, Cummins and co-workers disclosed the synthesis and reaction behavior of highly strained tri-*tert*-butylphosphatetrahedrane **20** (Scheme 17) [86]. Thus, upon the addition of BPh_3_ (20 mol%), **20** underwent rapid dimerization to form the bicyclic structure **22** in 72% yield. To trap potential intermediates, tetrahedrane **20** was treated with a 20-fold excess of styrene, which in the presence of BPh_3_ (20 mol%) furnished the cycloaddition product **23** in a yield of 88%. Similarly, BPh_3_ catalyzed the cycloaddition of **20** with 1 atm of ethylene to give the corresponding bicyclic structure **24** in yields of 74%. Quantum chemical calculations revealed BPh_3_ to mediate C−P bond cleavage to give intermediately tri-*tert*-butyl-phospacyclobutadiene **21**, which subsequently either dimerizes to **22** or undergoes a formal [2 + 4] cycloaddition with styrene and ethylene to yield **23** and **24**, respectively.

**Scheme 18:**
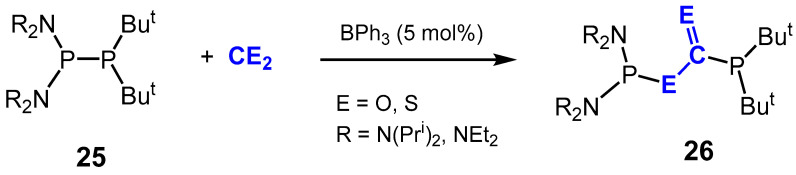
BPh_3_-catalyzed insertion of CE_2_ (E = O, S) into diphosphines.

Finally, Grubba et al. demonstrated the ability of BPh_3_ to act as an efficient Lewis acid catalyst in the diphosphination of CO_2_ and CS_2_ (Scheme 18) [87]. BPh_3_-catalyzed insertion of CO_2_ and CS_2_ into the P-P bond of unsymmetrical diphosphines **25** led to the selective formation of products **26** of the general formula (R_2_N)_2_P–E–C(=E)–P(Bu^t^)_2_, where the central carbon of CE_2_ binds to the more nucleophilic P(Bu^t^)_2_ moiety. In elucidating the reaction mechanism, it was found that neither CO_2_ nor the bulky diphosphines reacted with BPh_3_ individually to form the Lewis acid–base adducts, respectively, suggesting FLP-type behavior. It was proposed that the FLP diphosphine/BPh_3_ synergistically interacts with CO_2_ resulting in rapid P-P bond insertion.

## Data Availability

Not applicable.

## References

[1] Krause E., Nitsche R. (1922). Darstellung von Organischen Bor-Verbindungen Mit Hilfe von Borfluorid, II.: Bortriphenyl Und Phenyl-borsäure. Ber. Dtsch. Chem. Ges. A/B.

[2] Zettler F., Hausen H.D., Hess H. (1974). Crystal and Molecular Structure of Triphenylborane. J. Organomet. Chem..

[3] Guibert C.R., Little J.L. (2005). Alkyl- and Arylboranes. Ullmanns’s Encyclopedia of Industrial Chemistry.

[4] Wu Q., Esteghamatian M., Hu N.-X., Popovic Z., Enright G., Tao Y., D’Iorio M., Wang S. (2000). Synthesis, Structure, and Electroluminescence of BR_2_q (R = Et, Ph, 2-Naphthyl and q = 8-Hydroxyquinolato). Chem. Mater..

[5] Liu Q.-D., Mudadu M.S., Thummel R., Tao Y., Wang S. (2005). From blue to red: Syntheses, structures, electronic, and electroluminescent properties of tunable luminescent N,N chelate boron complexes. Adv. Funct. Mater..

[6] Chen H.-Y., Chi Y., Liu C.-S., Yu J.-K., Cheng Y.-M., Chen K.-S., Chou P.-T., Peng S.-M., Lee G.-H., Carty A.J. (2005). Rational color tuning and luminescent properties of functionalized boron-containing 2-pyridylpyrrolide complexes. Adv. Funct. Mater..

[7] Liddle B.J., Silva R.M., Morin T.J., Macedo F.P., Shukla R., Lindeman S.V., Gardinier J.R. (2007). BORAZANs: Tunable fluorophores based on 2-(pyrazolyl)aniline chelates of diphenylboron. J. Org. Chem..

[8] Tokoro Y., Nagai A., Kokado K., Chujo Y. (2009). Synthesis of Organoboron Quinoline-8-thiolate and Quinoline-8-selenolate Complexes and Their Incorporation into the π-Conjugated Polymer Main-Chain. Macromolecules.

[9] Li D., Wang K., Huang S., Qu S., Liu X., Zhu Q., Zhang H., Wang Y. (2011). Brightly fluorescent red organic solids bearing boron-bridged π-conjugated skeletons. J. Mater. Chem..

[10] Frath D., Azizi S., Ulrich G., Retailleau P., Ziessel R. (2011). Facile Synthesis of Highly Fluorescent Boranil Complexes. Org. Lett..

[11] Kubota Y., Hara H., Tanaka S., Funabiki K., Matsui M. (2011). Synthesis and Fluorescence Properties of Novel Pyrazine-Boron Complexes Bearing a β-Iminoketone Ligand. Org. Lett..

[12] Li D., Zhang H., Wang C., Huang S., Guo J., Wang Y. (2012). Construction of full-color-tunable and strongly emissive materials by functionalizing a boron-chelate four-ring-fused π-conjugated core. J. Mater. Chem..

[13] Shimizu S., Murayama A., Haruyama T., Iino T., Mori S., Furuta H., Kobayashi N. (2015). Benzo[c,d]indole-Containing Aza-BODIPY Dyes: Asymmetrization-Induced Solid-State Emission and Aggregation-Induced Emission Enhancement as New Properties of a Well-Known Chromophore. Chem. Eur. J..

[14] Wang X., Wu Y., Liu Q., Li Z., Yan H., Ji C., Duan J., Liu Z. (2015). Aggregation-induced emission (AIE) of pyridyl-enamido-based organoboron luminophores. Chem. Commun..

[15] Dou C., Ding Z., Zhang Z., Xie Z., Liu J., Wang L. (2015). Developing Conjugated Polymers with High Electron Affinity by Replacing a C-C Unit with a B-N Unit. Angew. Chem., Int. Ed..

[16] Liu F., Ding Z., Liu J., Wang L. (2017). An organoboron compound with a wide absorption spectrum for solar cell applications. Chem. Commun..

[17] Lu H., Nakamuro T., Yamashita K., Yanagisawa H., Nureki O., Kikkawa M., Gao H., Tian J., Shang R., Nakamura E. (2020). B/N-Doped p-Arylenevinylene Chromophores: Synthesis, Properties, and Microcrystal Electron Crystallographic Study. J. Am. Chem. Soc..

[18] Krause E. (1924). Valence problem of boron: A series of strikingly stable secondary valence compounds of boron triphenyl. Ber. Dtsch. Chem. Ges..

[19] Nakamura T., Umeno M. (2000). Triarylborane-Amine Compounds, and Their (co)Polymers, and Antifouling Agents. Japan Patent.

[20] Shimada A., Kohara M., Shibuya Y. (1998). Triphenylboron-Containing Polymers and Their Use as Marine Antifouling Agents.

[21] Saeki Y., Kumagaya H. (1998). Synergistic Marine Antifouling Agents Containing Triphenylboranes and Higher Aliphatic Polyamines. Japan Patent.

[22] Lawson K.R., Mound W.R., Whittingham W.G. (1997). Preparation of Pyrazolylborane Derivatives as Fungicides in Plants. World Intellectual Property Organization.

[23] Lawson K.R., Mound W.R., Whittingham W.G. (1997). Preparation of Pyrimidinylborane Derivatives as Fungicides. World Intellectual Property Organization.

[24] Tsang T.H., Strutzel J.L. (1991). Preparation of Bis(amineborane)alkanes as Agrochemical Fungicides. U.S. Patent.

[25] Massey A.G., Park A.J., Stone F.G.A. (1963). Tris(pentafluorophenyl)boron. Proc. Chem. Soc..

[26] Mayer R.J., Hampel N., Ofial A.R. (2021). Lewis Acidic Boranes, Lewis Bases, and Equilibrium Constants: A Reliable Scaffold for a Quantitative Lewis Acidity/Basicity Scale. Chem. Eur. J..

[27] Gao H., Battley A., Leitao E.M. (2022). The Ultimate Lewis Acid Catalyst: Using Tris(Pentafluorophenyl) Borane to Create Bespoke Siloxane Architectures. Chem. Commun..

[28] Nori V., Pesciaioli F., Sinibaldi A., Giorgianni G., Carlone A. (2022). Boron-Based Lewis Acid Catalysis: Challenges and Perspectives. Catalysts.

[29] Kumar G., Roy S., Chatterjee I. (2021). Tris(Pentafluorophenyl)Borane Catalyzed C–C and C–Heteroatom Bond Formation. Org. Biomol. Chem..

[30] Hackel T., McGrath N.A. (2019). Tris(Pentafluorophenyl)Borane-Catalyzed Reactions Using Silanes. Molecules.

[31] Park S. (2019). B(C_6_F_5_)_3_-Catalyzed Sp^3^ C—Si Bond Forming Consecutive Reactions. Chin. J. Chem..

[32] Lawson J.R., Melen R.L. (2017). Tris(Pentafluorophenyl)Borane and Beyond: Modern Advances in Borylation Chemistry. Inorg. Chem..

[33] Oestreich M., Hermeke J., Mohr J. (2015). A Unified Survey of Si–H and H–H Bond Activation Catalysed by Electron-Deficient Boranes. Chem. Soc. Rev..

[34] Brook M.A., Grande J.B., Ganachaud F., Muzafarov A.M. (2010). New Synthetic Strategies for Structured Silicones Using B(C_6_F_5_)_3_. Silicon Polymers.

[35] Erker G. (2005). Tris(Pentafluorophenyl)Borane: A Special Boron Lewis Acid for Special Reactions. Dalton Trans..

[36] Piers W.E., Chivers T. (1997). Pentafluorophenylboranes: From Obscurity to Applications. Chem. Soc. Rev..

[37] Welch G.C., Juan R.R.S., Masuda J.D., Stephan D.W. (2006). Reversible, Metal-Free Hydrogen Activation. Science.

[38] Welch G.C., Stephan D.W. (2007). Facile Heterolytic Cleavage of Dihydrogen by Phosphines and Boranes. J. Am. Chem. Soc..

[39] Spies P., Erker G., Kehr G., Bergander K., Fröhlich R., Grimme S., Stephan D.W. (2007). Rapid Intramolecular Heterolytic Dihydrogen Activation by a Four-Membered Heterocyclic Phosphane–Borane Adduct. Chem. Commun..

[40] Mummadi S., Brar A., Wang G., Kenefake D., Diaz R., Unruh D.K., Li S., Krempner C. (2018). “Inverse” Frustrated Lewis Pairs: An Inverse FLP Approach to the Catalytic Metal Free Hydrogenation of Ketones. Chem. Eur. J..

[41] Paradies J. (2014). Metal-Free Hydrogenation of Unsaturated Hydrocarbons Employing Molecular Hydrogen. Angew. Chem. Int. Ed..

[42] Farrell J.M., Posaratnanathan R.T., Stephan D.W. (2015). A Family of N-Heterocyclic Carbene-Stabilized Borenium Ions for Metal-Free Imine Hydrogenation Catalysis. Chem. Sci..

[43] Erős G., Mehdi H., Pápai I., Rokob T.A., Király P., Tárkányi G., Soós T. (2010). Expanding the Scope of Metal-Free Catalytic Hydrogenation through Frustrated Lewis Pair Design. Angew. Chem. Int. Ed..

[44] Xu B.-H., Kehr G., Fröhlich R., Wibbeling B., Schirmer B., Grimme S., Erker G. (2011). Reaction of Frustrated Lewis Pairs with Conjugated Ynones-Selective Hydrogenation of the Carbon-Carbon Triple Bond. Angew. Chem. Int. Ed..

[45] Chernichenko K., Madarász Á., Pápai I., Nieger M., Leskelä M., Repo T. (2013). A Frustrated-Lewis-Pair Approach to Catalytic Reduction of Alkynes to *Cis*-Alkenes. Nature Chem..

[46] Kobayashi M., Sanda F., Endo T. (2001). Application of (Triphenylphosphinemethyl-Ene)Boranes to Thermally Latent Catalysts for Polyaddition of Bisphenol A Diglycidyl Ether with Bisphenol A: Model System of Epoxy−Novolac Resin. Macromolecules.

[47] Kobayashi M., Sanda F., Endo T. (2002). Substituent Effect of (Triphenylphosphinemethylene)Boranes on Latent Catalytic Activity for Polyaddition of Bisphenol A Diglycidyl Ether with Bisphenol A: Model System of Epoxy−Novolac Resin. Macromolecules.

[48] Wang L., Kodamaa K., Hirose T. (2016). DBU/benzyl bromide: An efficient catalytic system for the chemical fixation of CO2 into cyclic carbonates under metal- and solvent-free conditions. Catal. Sci. Technol..

[49] Wang L., Zhang G., Kodamaa K., Hirose T. (2016). An efficient metal- and solvent-free organocatalytic system for chemical fixation of CO_2_ into cyclic carbonates under mild conditions. Green Chem..

[50] Zhang D., Boopathi S.K., Hadjichristidis N., Gnanou Y., Feng X. (2016). Metal-Free Alternating Copolymerization of CO_2_ with Epoxides: Fulfilling “Green” Synthesis and Activity. J. Am. Chem. Soc..

[51] Yang J.-L., Wu H.-L., Li Y., Zhang X.-H., Darensbourg D.J. (2017). Perfectly Alternating and Regioselective Copolymerization of Carbonyl Sulfide and Epoxides by Metal-Free Lewis Pairs. Angew. Chem. Int. Ed..

[52] Andrea K.A., Kerton F.M. (2019). Triarylborane-Catalyzed Formation of Cyclic Organic Carbonates and Polycarbonates. ACS Catal..

[53] Andrea K.A., Kerton F.M. (2019). Functionalized Polycarbonates via Triphenylborane Catalyzed Polymerization-Hydrosilylation. RSC Adv..

[54] Andrea K.A., Wheeler M.D., Kerton F.M. (2021). Borane Catalyzed Polymerization and Depolymerization Reactions Controlled by Lewis Acidic Strength. Chem. Commun..

[55] Jia M., Hadjichristidis N., Gnanou Y., Feng X. (2021). Polyurethanes from Direct Organocatalytic Copolymerization of *p* -Tosyl Isocyanate with Epoxides. Angew. Chem. Int. Ed..

[56] Stephan D.W., Bullock R.M. (2010). Catalysis without Precious Metals.

[57] Stephan D.W., Greenberg S., Graham T.W., Chase P., Hastie J.J., Geier S.J., Farrell J.M., Brown C.C., Heiden Z.M., Welch G.C. (2011). Metal-Free Catalytic Hydrogenation of Polar Substrates by Frustrated Lewis Pairs. Inorg. Chem..

[58] Stephan D.W. (2012). “Frustrated Lewis Pair” Hydrogenations. Org. Biomol. Chem..

[59] Erker G., Stephan D.W. (2013). Frustrated Lewis Pairs I: Uncovering and Understanding.

[60] Hounjet L.J., Stephan D.W. (2014). Hydrogenation by Frustrated Lewis Pairs: Main Group Alternatives to Transition Metal Catalysts?. Org. Process Res. Dev..

[61] Rokob T.A., Hamza A., Pápai I. (2009). Rationalizing the Reactivity of Frustrated Lewis Pairs: Thermodynamics of H _2_ Activation and the Role of Acid−Base Properties. J. Am. Chem. Soc..

[62] Li H., Aquino A.J.A., Cordes D.B., Hung-Low F., Hase W.L., Krempner C. (2013). A Zwitterionic Carbanion Frustrated by Boranes—Dihydrogen Cleavage with Weak Lewis Acids via an “Inverse” Frustrated Lewis Pair Approach. J. Am. Chem. Soc..

[63] Miller A.J.M., Labinger J.A., Bercaw J.E. (2010). Homogeneous CO Hydrogenation: Dihydrogen Activation Involves a Frustrated Lewis Pair Instead of a Platinum Complex. J. Am. Chem. Soc..

[64] Mummadi S., Unruh D.K., Zhao J., Li S., Krempner C. (2016). “Inverse” Frustrated Lewis Pairs—Activation of Dihydrogen with Organosuperbases and Moderate to Weak Lewis Acids. J. Am. Chem. Soc..

[65] Geng J., Wu Y., Hu X. (2021). Controlling the Lewis Acidity and Polymerizing Effectively Prevent Frustrated Lewis Pairs from Deactivation in the Hydrogenation of Terminal Alkynes. Org. Lett..

[66] Tsushima D., Igarashi M., Sato K., Shimada S. (2017). Ir-Catalyzed Hydrogenolysis Reaction of Silyl Triflates and Halides with H_2_. Chem. Lett..

[67] Beppu T., Sakamoto K., Nakajima Y., Matsumoto K., Sato K., Shimada S. (2018). Hydrosilane Synthesis via Catalytic Hydrogenolysis of Halosilanes Using a Metal-Ligand Bifunctional Iridium Catalyst. J. Organomet. Chem..

[68] Glüer A., Schweizer J.I., Karaca U.S., Würtele C., Diefenbach M., Holthausen M.C., Schneider S. (2018). Hydrosilane Synthesis by Catalytic Hydrogenolysis of Chlorosilanes and Silyl Triflates. Inorg. Chem..

[69] Durin G., Berthet J.-C., Nicolas E., Thuéry P., Cantat T. (2022). The Role of (*^t^*BuPOCOP)Ir(I) and Iridium(III) Pincer Complexes in the Catalytic Hydrogenolysis of Silyl Triflates into Hydrosilanes. Organometallics.

[70] Durin G., Berthet J.-C., Nicolas E., Cantat T. (2021). Unlocking the Catalytic Hydrogenolysis of Chlorosilanes into Hydrosilanes with Superbases. ACS Catal..

[71] Durin G., Fontaine A., Berthet J., Nicolas E., Thuéry P., Cantat T. (2022). Metal-Free Catalytic Hydrogenolysis of Silyl Triflates and Halides into Hydrosilanes. Angew. Chem. Int. Ed..

[72] Kira M., Müller T. (2017). Dihydrogen Splitting Using Dialkylsilylene-Based Frustrated Lewis Pairs. Chem. Asian J..

[73] Li Y., Molina de La Torre J.A., Grabow K., Bentrup U., Junge K., Zhou S., Brückner A., Beller M. (2013). Selective Reduction of Amides to Amines by Boronic Acid Catalyzed Hydrosilylation. Angew. Chem. Int. Ed..

[74] Chadwick R.C., Kardelis V., Lim P., Adronov A. (2014). Metal-Free Reduction of Secondary and Tertiary *N* -Phenyl Amides by Tris(Pentafluorophenyl)Boron-Catalyzed Hydrosilylation. J. Org. Chem..

[75] Blondiaux E., Cantat T. (2014). Efficient Metal-Free Hydrosilylation of Tertiary, Secondary and Primary Amides to Amines. Chem. Commun..

[76] Mukherjee D., Shirase S., Mashima K., Okuda J. (2016). Chemoselective Reduction of Tertiary Amides to Amines Catalyzed by Triphenylborane. Angew. Chem. Int. Ed..

[77] Mukherjee D., Sauer D.F., Zanardi A., Okuda J. (2016). Selective Metal-Free Hydrosilylation of CO_2_ Catalyzed by Triphenylborane in Highly Polar, Aprotic Solvents. Chem. Eur. J..

[78] Berkefeld A., Piers W.E., Parvez M. (2010). Tandem Frustrated Lewis Pair/Tris(Pentafluorophenyl)Borane-Catalyzed Deoxygenative Hydrosilylation of Carbon Dioxide. J. Am. Chem. Soc..

[79] Murata T., Hiyoshi M., Maekawa S., Saiki Y., Ratanasak M., Hasegawa J., Ema T. (2022). Deoxygenative CO_2_ Conversions with Triphenylborane and Phenylsilane in the Presence of Secondary Amines or Nitrogen-Containing Aromatics. Green Chem..

[80] Ratanasak M., Murata T., Adachi T., Hasegawa J., Ema T. (2022). Mechanism of BPh_3_-Catalyzed N-Methylation of Amines with CO_2_ and Phenylsilane: Cooperative Activation of Hydrosilane. Chem. A Eur. J..

[81] Scott D.J., Simmons T.R., Lawrence E.J., Wildgoose G.G., Fuchter M.J., Ashley A.E. (2015). Facile Protocol for Water-Tolerant “Frustrated Lewis Pair”-Catalyzed Hydrogenation. ACS Catal..

[82] Fasano V., Ingleson M.J. (2017). Expanding Water/Base Tolerant Frustrated Lewis Pair Chemistry to Alkylamines Enables Broad Scope Reductive Aminations. Chem. Eur. J..

[83] Fasano V., Radcliffe J.E., Ingleson M.J. (2016). B(C_6_F_5_)_3_-Catalyzed Reductive Amination Using Hydrosilanes. ACS Catal..

[84] Brar A., Unruh D.K., Ling N., Krempner C. (2019). BPh_3_-Catalyzed [2+3] Cycloaddition of Ph_3_PCCO with Aldonitrones: Access to 5-Isoxazolidinones with Exocyclic Phosphonium Ylide Moieties. Org. Lett..

[85] Brar A., Unruh D.K., Aquino A.J., Krempner C. (2019). Lewis Acid Base Chemistry of Bestmann’s Ylide, Ph_3_PCCO, and Its Bulkier Analogue, (Cyclohexyl)_3_PCCO. Chem. Commun..

[86] Riu M.-L.Y., Eckhardt A.K., Cummins C.C. (2021). Dimerization and Cycloaddition Reactions of Transient Tri- *Tert* -Butylphosphacyclobutadiene Generated by Lewis Acid Induced Isomerization of Tri- *Tert* -Butylphosphatetrahedrane. J. Am. Chem. Soc..

[87] Szynkiewicz N., Ponikiewski Ł., Grubba R. (2019). Diphosphination of CO_2_ and CS_2_ Mediated by Frustrated Lewis Pairs—Catalytic Route to Phosphanyl Derivatives of Formic and Dithioformic Acid. Chem. Commun..

